# Improving SARS-CoV-2 structures: Peer review by early coordinate release

**DOI:** 10.1016/j.bpj.2020.12.029

**Published:** 2021-01-16

**Authors:** Tristan I. Croll, Christopher J. Williams, Vincent B. Chen, David C. Richardson, Jane S. Richardson

**Affiliations:** 1 CIMR, University of Cambridge, Cambridge, United Kingdom; 2 Department of Biochemistry, Duke University, Durham, North Carolina

## Abstract

This work builds upon the record-breaking speed and generous immediate release of new experimental three-dimensional structures of the severe acute respiratory syndrome coronavirus 2 (SARS-CoV-2) proteins and complexes, which are crucial to downstream vaccine and drug development. We have surveyed those structures to catch the occasional errors that could be significant for those important uses and for which we were able to provide demonstrably higher-accuracy corrections. This process relied on new validation and correction methods such as CaBLAM and ISOLDE, which are not yet in routine use. We found such important and correctable problems in seven early SARS-CoV-2 structures. Two of the structures were soon superseded by new higher-resolution data, confirming our proposed changes. For the other five, we emailed the depositors a documented and illustrated report and encouraged them to make the model corrections themselves and use the new option at the worldwide Protein Data Bank for depositors to re-version their coordinates without changing the Protein Data Bank code. This quickly and easily makes the better-accuracy coordinates available to anyone who examines or downloads their structure, even before formal publication. The changes have involved sequence misalignments, incorrect RNA conformations near a bound inhibitor, incorrect metal ligands, and *cis-trans* or peptide flips that prevent good contact at interaction sites. These improvements have propagated into nearly all related structures done afterward. This process constitutes a new form of highly rigorous peer review, which is actually faster and more strict than standard publication review because it has access to coordinates and maps; journal peer review would also be strengthened by such access.

## Significance

Accurate three-dimensional structures of macromolecules from the severe acute respiratory syndrome coronavirus 2 virus provide information essential for understanding the biology of the virus and for rapid and effective design of vaccines and drugs to combat the pandemic. We used new validation and correction techniques on the early-release severe acute respiratory syndrome structures, sometimes finding significant local errors. We contacted depositors, encouraging them to make the corrections themselves using the new versioning system at the worldwide Protein Data Bank (PDB), which lets them improve all future downloads without changing their PDB code. This has enabled many local corrections, including sequence alignment, RNA base pairing, metal ligands, and backbone conformations; the better accuracy propagates into related later structures. This constitutes a new paradigm for very rapid and unusually rigorous peer review.

## Introduction

In this truly urgent crisis of the coronavirus disease 2019 (COVID-19) pandemic, the worldwide research community has mobilized to provide amazingly rapid understanding of the biology of the severe acute respiratory syndrome coronavirus 2 (SARS-CoV-2) virus and many new paths toward its possible control. As early as February, often in broad collaborations, structural biologists had begun to deposit structures of the proteins and their complexes from the new virus. In a break with tradition, these structures are being released to the public immediately, which in turn greatly speeds downstream research and development.

Early-release structures have not yet gone through all the cross-checks involved in writing and reviewing a formal article, so it is understandable they will contain somewhat more mistakes. However, most parts of these structures are up to the standards expected for their resolution and local degree of order, and often the overall molecular arrangement can provide quite unexpected and valuable new insights. For instance, in PDB: 6w41, an antibody that blocks spike binding to the ACE2 receptor interacts with a nonoverlapping part of the spike protein’s surface, and in PDB: 6zm7, the viral Nsp1 protein inhibits a cell’s antiviral defenses by stuffing itself into the messenger RNA channel of human ribosomes to prevent synthesis of the defense proteins (1).

In contrast, a more detailed and nuanced use of a structure, such as creating or modifying high-specificity binding molecules to produce an effective drug or vaccine, can be chancy from early-release structures and benefits greatly from the best feasible accuracy of conformation and atom placement in the relevant contact area. Therefore, a number of groups that specialize in validating and correcting three-dimensional macromolecular structures have been concentrating on the new SARS-CoV-2 depositions. Andrea Thorn has gathered an extremely broad set of experts to form the Coronavirus Structural Task Force (CSTF). Their website (http://github.com/thorn-lab/coronavirus_structural_task_force/) brings together a variety of information on all the hundreds of SARS-CoV-2 and related structures, validation reports from several programs (now including MolProbity), rebuilt models from several sources, and information about the virus biology as outreach to the public (2). The http://covid-19.bioreproducibility.org/ website hosts rebuilt structures with a concentration on the important aspect of bound ligands (3). The PDB-Redo site http://www.cmbi.ru.nl/pdb_redo/ has for a number of years routinely done re-refinement and automated local corrections for all PDB entries, and that of course continues for the new SARS-CoV-2 structures (4). These, and ours, are probably not the only such efforts.

The authors of this article have worked more behind the scenes, to get the clearest and most important corrections to SARS-CoV-2 models updated directly in the PDB by the depositors themselves without changing PDB code, possible since the new versioning system announced in the PDB News item of July 31, 2019. Now that most formal publications based on those re-versioned structures are out, we are here describing our strategies and the available but not yet mainstream methods that made this possible, with much of it being visual and interactive.

## Methods

Structures of SARS-CoV-2 macromolecules were identified by searches at the RCSB or PDBe sites of the worldwide Protein Data Bank (wwPDB; (5)) and by entries on the CSTF website (2). Because other groups are concentrating on the bound ligands, and because our expertise is in conformational analysis of protein and RNA three-dimensional structures, we prioritized cases in which those conformations and binding surfaces are likely to matter for understanding the virus biology and host interactions or for drug and vaccine design. Coordinates and density maps were downloaded from the PDB or the Electron Microscopy Database ((6). For crystal structures, we used 2mF_obs_-dF_calc_ and F_obs_-F_calc_ difference maps, and for cryo-EM structures, we used the primary map and only occasionally a focused map. In KiNG, usually two interactively adjusted contour levels were visualized, with the lower in gray and the higher in black. In ISOLDE, the original map is shown as a transparent surface, sometimes with a wireframe overlay of a map desharpened by B = +50.

The SARS-CoV-2 structures were surveyed for possible problems in two complementary ways: by running MolProbity validation (7) and by initial behavior when starting up molecular dynamics in ISOLDE (8). Both of those validations are automated, as is generally true for validations; the only exceptions here were the Zn^2+^ for Cl^−^ in PDB: 6vy0 and the half-occupancy remdesivir in PDB: 7bv2. A central, and still unique, aspect of MolProbity is all-atom contact analysis, which uses the Reduce program (9) to add and optimize H atoms and then the Probe program (10) to measure the nonpairwise surface contacts between all atoms in the model. It outputs an overall “clashscore” evaluation, and most importantly for this application, it provides quantitative data and visual markup for local H-bonds, van der Waals contacts, and serious clashes (defined as overlaps ≥0.4 Å). For RNA, MolProbity provides criteria for ribose pucker and backbone conformers (11). MolProbity’s version of traditional validations include outliers in bond lengths and angles, Ramachandran values, and side-chain rotamers. These are still extremely effective at resolutions better than ∼2.5 Å and do flag problems whenever they occur, but at lower resolutions, they are very often not seen because they have been tightly restrained to achieve stable, convergent refinement, usually without fixing the underlying problems (12).

Because tight restraints to traditional validation criteria have destroyed their usefulness at resolutions poorer than 2.5 Å, new validation criteria are badly needed that can still provide meaningful assessment in that regime. Because model building does not yet use Bayesian likelihood to trade off conformational probability with density fit, very rare conformations can be greatly overused at low resolutions or in regions of poor density. For instance, for the case of *cis*-nonPro peptides (which occur genuinely in only 1 out of 3000 residues), that problem was of epidemic proportions for ∼10 years (13); they are now strongly flagged in MolProbity (14) and elsewhere, and unjustifiable *cis*-nonPro are back to much lower levels.

So far, the most generally applicable MolProbity tool for 2.5–4 Å is CaBLAM (7,15), which uses C*α* virtual angles to determine a robust backbone trace and then a virtual angle between successive backbone CO bond directions to find where peptide orientations are not compatible with the local C*α* trace. CaBLAM flags incorrect peptide orientations even when Ramachandran outliers have been refined away, and in the recent cryo-EM model challenge, the CaBLAM score was found to have a higher correlation with match to target than any other criterion (12). In development is RNAprecis, a criterion to improve both modeling and validation of full-detail RNA conformations using features visible even at 3.5 Å.

Corrections to outliers are almost always done by a visual, interactive combination of user-driven control of computational procedures. We examined as many outliers as feasible, prioritizing them in two ways: first, outliers in important areas such as active sites, bound ions or ligands, between chains or molecules, or where known conformational changes occur; second, outliers where prior probability plus local map, fit, and contact quality are sufficient to distinguish clearly between specific proposed alternative interpretations. At a resolution of 3–4 Å, especially in large structures, we have found there are three tiers of certainty versus uncertainty. 1) In the best parts, usually the central core, the map is usually clear enough to determine an unambiguous model fitting, with only occasional definable errors. 2) There are always some mobile regions with such low local resolution that they show density but do not determine a single model, where in most cases no alternative can be reliably judged as best. 3) In between those extremes, we concentrate on identifying and correcting problems for which we can clearly document by multiple criteria that the suggested corrections are genuine improvements.

Much of the examination was done in KiNG interactive graphics (16), which shows model, map, and all MolProbity markup; KiNG is very good at side-chain correction coupled with subtle “backrub” backbone shifts (17), and can make limited further backbone changes. COOT (18) could quite often correct CaBLAM outliers, although it does not yet display their markup. The most general system for interactive correction was ISOLDE, which is described below. After a model had been corrected, it was briefly re-refined in PHENIX (19). Efforts are underway to automate correcting at least some classes of lower-resolution outliers, but so far, they have not succeeded nearly as well as manual correction.

Rebuilding in ISOLDE is accomplished via repeated local interactive molecular dynamics simulations biased by the experimental density map. ISOLDE runs as a plug-in to ChimeraX (20) using a molecular dynamics flexible fitting (21) approach. Each simulation is typically on the scale of a few dozen to a few hundred amino acid residues—large enough to remodel a problem region but small enough to support simulation speeds sufficient for interaction. Remodeling is accomplished via the combination of direct user tugging with scripted tools for common tasks such as *cis-trans* change of peptide geometry, flipping of peptide orientation, adjusting of rotamers, or shifting a selected stretch in sequence register. The Ramachandran and rotamer quality of each residue is marked up in real time as the model evolves; restraints are not used for Ramachandran or rotamers, with the rare exception of individual rotamer restraints applied by the user on a case-by-case basis. As is typical of molecular dynamics simulations, for a model settled in ISOLDE, the clashscore within an individual asymmetric unit is always close to zero (although severe clashes with symmetry neighbors remain possible) because of the explicit modeling of the van der Waals potential. Clashing atoms are instead pushed out of density, usually leading to easier diagnosis and correction of the underlying problem. Each model was inspected and remodeled at least once, residue by residue and end to end, in overlapping simulations in ISOLDE. Crystal structures were then refined in phenix.refine to obtain the benefits of phase optimization, with the model acting as its own reference for the purpose of torsion restraints. For cryo-EM structures, a second map smoothed with B+50 was sometimes overlaid in wireframe; the simulations feel contributions from both maps, which can improve convergence. For lower-resolution (>3 Å) data sets, the resulting models were rebuilt and re-refined one to two more times. The most significant changes were noted and prioritized by at least two different people.

When important local errors were identified and convincingly corrected, we emailed the depositor with explanations and illustrations of those changes. We included a revised coordinate file but encouraged them to make and confirm the changes themselves and to use the new wwPDB versioning system to update their deposited structure quickly and easily, usually in these cases before formal publication (see the last section in Results).

As well as the revised coordinate files from ISOLDE that are posted on the CSTF website, our team has worked with the CSTF to provide simplified but complete MolProbity validation output for all the severe-acute-respiratory-syndrome-related structures. This was enabled by revising MolProbity’s Ramachandran and Cbeta deviation PDF outputs to work better with very large structures and fitting the overall information into file-size limits on the GitHub site.

Fig. 8 was made in ISOLDE by Tristan Croll to represent a close-up of how problems were seen and corrected in that highly complex and real-time interactive environment. The rest were made in KiNG by the Richardsons, in which CaBLAM outlier corrections can be shown and made, and which has more facilities for two-dimensional static presentation graphics at a variety of scales.

We use a convention for PDB codes that prevents 1, l, I or O, 0 ambiguity in any font: letters are in lower case except for L, as in PDB: 6yLa.

## Results and discussion

### RNA-binding nucleocapsid phosphoprotein

**Figure 1:**
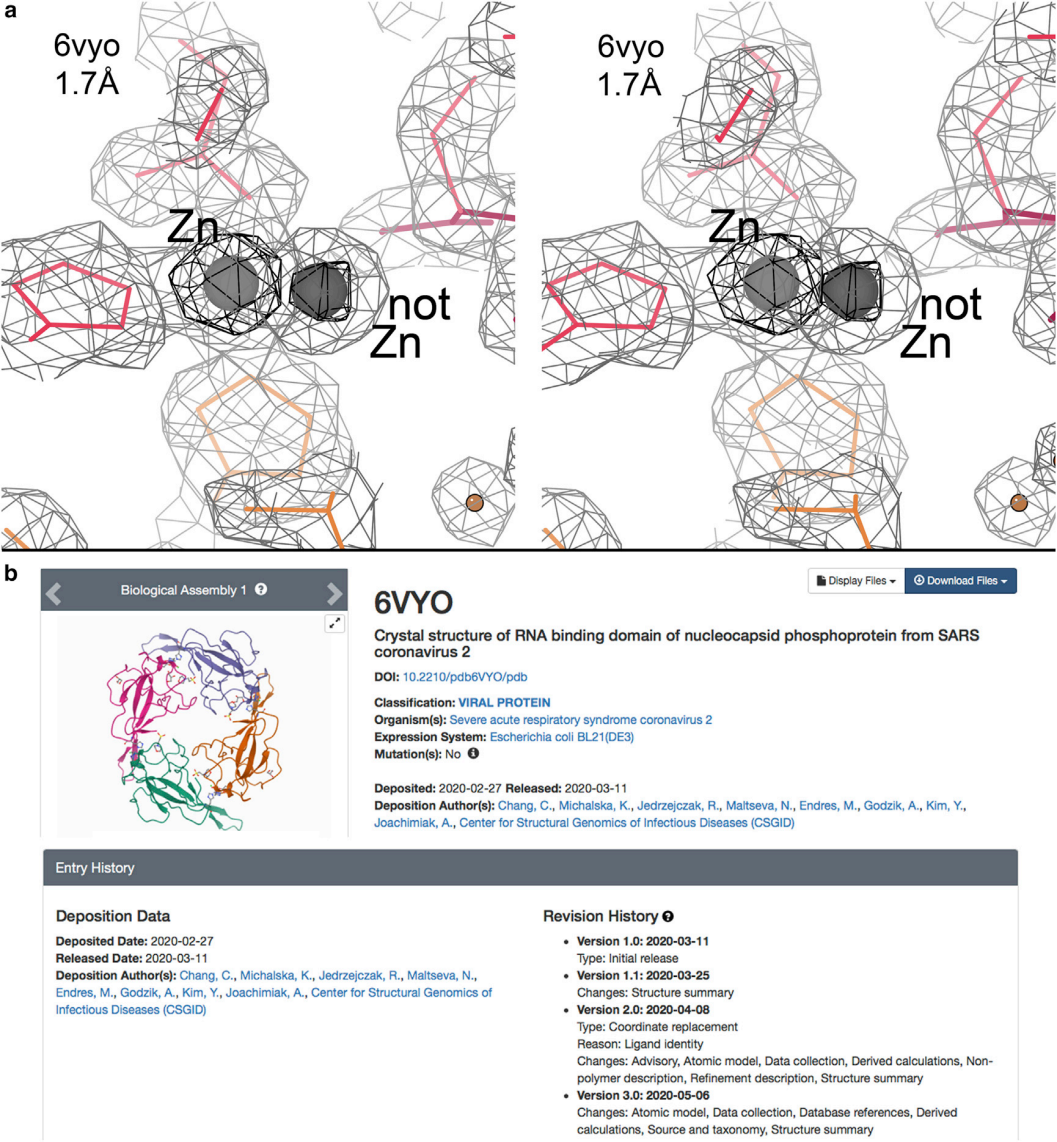
(*a*) Stereo of the original too-close zinc ions in 6vyo; the outer, lower-density ion is really Cl^–^. (*b*) Revision history on the RCSB page for 6vyo, where version 2.0 lists coordinate replacement for ligand identity on 2020-04-08. To see this figure in color, go online.

Our first example from SARS-CoV-2 was file PDB: 6vyo, deposited on February 27, 2020, and first released on March 11. It is a 1.7-Å x-ray structure of the tetrameric RNA-binding phosphoprotein of the internal nucleocapsid that holds the viral genome. Overall, it is an excellent structure with a highly interpretable map and very few validation outliers. But visual inspection of model and map in KiNG, starting at the important zinc site in each subunit, showed a chemically implausible, partially occupied second Zn^2+^ only 2.2 Å from the primary Zn^2+^ and positioned as one of its four tetrahedral ligands, all with clean electron density (see stereo Fig. 1 *a*). This second ion has the wrong charge for its position. Fortunately, crystallization conditions were reported in the PDB file to include ZnCl_2_, so almost certainly these secondary sites are full-occupancy Cl^−^. This incorrect atom identity in the 6vyo model was presumably an accidental oversight and is very straightforward to correct.

The Richardsons emailed Andrzej Joachimiak, the depositor of record, on March 24, describing the problem and the easy route for him to re-version the coordinates at the wwPDB. He replied the next day, saying that he agreed and would change it, and the new version 2.0 was released on April 8, less than 2 weeks later. Since then, anyone who downloads 6vyo from any wwPDB site automatically gets the improved coordinates. Along with the PDB’s just-in-time depositor-initiated re-versioning system instituted last fall, the complete version history is now made obvious, as seen for the instance in Fig. 1 *b*, at bottom right of the 6vyo RCSB-PDB web page at http:/www.rcsb.org/pdb (see PDB: 6vyo).

This inadvertent error was the structural equivalent of a “typo,” but one that changed the meaning in an important location. It is a rare and unexpected type of error not tested by automated structure validation or fixed by refinement or by PDB-Redo (4).

As well as our visual inspection, we later learned that the interactive molecular dynamics of Tristan Croll’s ISOLDE program had also strongly flagged this problem, and Croll had posted his revised structure on the CSTF website. Going forward, he joined our collaborative group, which has so far resulted in the work described here.

### Spike receptor-binding domain/antibody

Our second SARS-CoV-2 example was 6w41 at 3.08 Å, an antibody bound to the spike protein’s RBD (Receptor Binding Domain). Surprisingly, although the antibody is a nanomolar binder and prevents ACE2 binding in vitro, it interacts with a nonoverlapping part of the RBD.

**Figure 2:**
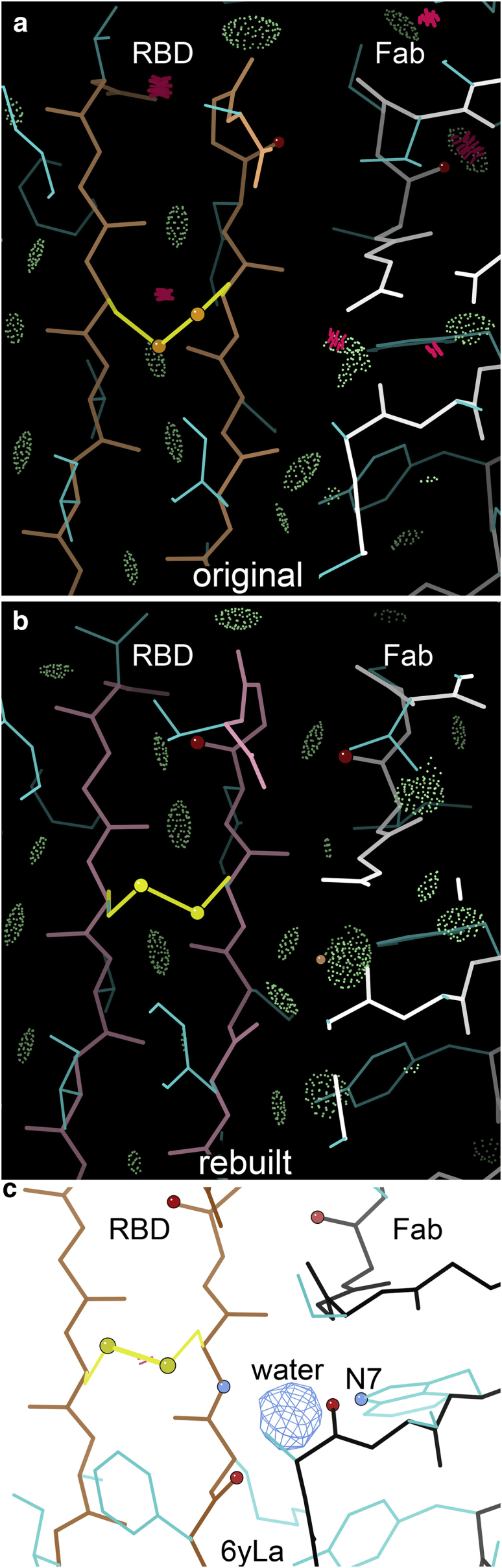
Interface of spike RBD and Fab CR3022. (*a*) In 6w41 at 3.08 Å, with strained SS (*yellow*), six clashes (*red spikes*), and two peptide flips (*red ball* on CO). (*b*) Rebuilt, with five interface H-bonds (*green dots*) and a proposed bridging water. (*c*) In 6yLa at 2.42 Å, confirming SS staple, peptide flips, and water (*blue* difference-map contours). To see this figure in color, go online.

The core of that interface is a six-residue edge *β*-strand, with its center disulfide-linked to the neighbor strand in the sheet (Fig. 2 *a*). Such a link, called an SS staple, has only one possible conformation, with **−**, **−**, **+**, **−**,**−** dihedral angles (22). The 6w41 model has a highly strained SS conformation with clashing H*α*’s and **t**, 120°, **−**, 0°, **−** dihedrals, two of which are eclipsed. That SS seems to distort the edge *β*-strand, resulting in two flipped peptides, six bad clashes, and very poor contact between the RBD and Fab, with only one H-bond and sparse van der Waals contact. In addition, several of the *N*-acetyl glucosamine (NAG) carbohydrates were fit backward at the bond to the protein.

Our rebuilt version (Fig. 2 *b*) has the standard SS staple conformation and corrects the peptides to give H-bonds instead of clashes. Trp33 H-chain, seen edge-on at right, is buried in the contact but too far from the RBD to satisfy its ring N7 (Ne1) atom. We modeled a water (small orange ball) not seen at this resolution but in position to bridge the gap with 4 tetrahedral H-bonds, made some rotamer changes, and added several ions at the surface. We contacted the authors, but they were already close to finishing a new structure at 2.42 Å: 6yLa. As shown in Fig. 2 *c*, it confirmed all the major corrections—the SS staple conformation and the correctly oriented, H-bonding peptides—and even showed a clear positive difference peak at the proposed water position.

In another case, better new data also soon became available that corrected the major problems—always the preferred outcome. PDB: 6w9c at 2.7 Å is the papain-like protease of SARS-Cov-2. The long arms of the trimer end in zinc finger domains important to one phase of the activity, but their cryo-EM density is very poorly resolved. The three Zn sites were modeled independently, with ligands missing, misoriented, or even SS linked. We could improve the model somewhat, and another team of the Coronavirus Structural Task Force reprocessed the data and also improved the model somewhat (Croll 2020), but the new mutant structure at 1.6 Å (PDB: 6wrh) really solved the problem satisfactorily.

### RNA-dependent RNA polymerase: Nsp12/Nsp8/Nsp7 complex

The Nsp12 RNA polymerase, with its helper proteins Nsp7 and 8, is essential for replication of the SARS-CoV-2 viral genome. The first structures of this complex were PDB: 6m71 at 2.9 Å and PDB: 7btf at 2.95 Å by cryo-EM (23). We chose to work most intensively on 7btf because inclusion of DTT prevented SS formation and preserved the biological Zn sites; however, most of the same problems also occur in the 6m71 and PDB: 7bv1 structures of this complex.

#### Sequence +1 register shift at the nsp8 (chB) to nsp12 interface

**Figure 3:**
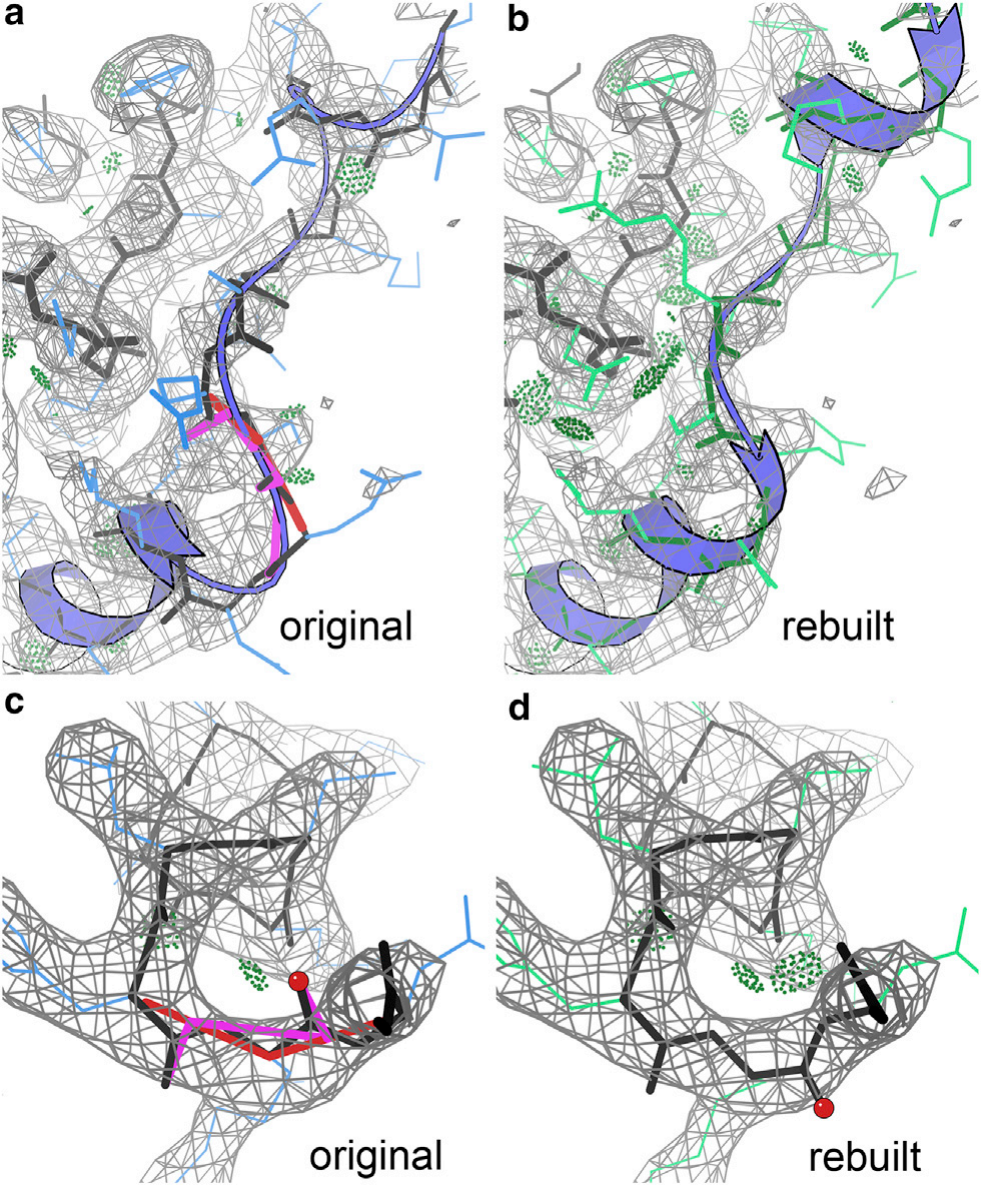
Corrections in 7btf. (*a*) Nsp8 (chain B, with *ribbon*) N-terminal +1 sequence offset produces CaBLAM outliers where it distorts to get back in register at Lys 79. (*b*) Rebuilt model has a classic helix N-cap and no outliers. Density contours at 5.3*σ*. (*c*) Peptide A733-4 at the end of a regular *α*-helix has CaBLAM and C*α* geometry outliers. (*d*) With peptide flipped, now a good helix C-cap. Density contours at 12*σ*. To see this figure in color, go online.

This problem was discovered and rebuilt in ISOLDE and was confirmed using MolProbity’s CaBLAM and all-atom-contact functions (7) along with examining by eye the fit of model to map. The chain B N-terminal dozen visible residues are misaligned by +1 until joining correctly at Lys79 in the first helix. Two CaBLAM outliers (magenta) and a C*α* geometry outlier (red) flag the unlikely backbone conformation caused by squeezing in the extra residue, as shown in Fig. 3 *a*. CaBLAM flags peptide CO orientations not compatible with the local C*α* trace (15). Fig. 3 *b* shows the rebuilt section in correct sequence register, with no CaBLAM outliers, a normal helix N-cap, and much better H-bonding and contact with Nsp12. Backbone fit is a bit better in the corrected version, especially at the helix start, but local map density is rather low and patchy. Sequence in the misaligned section (MTQMYKQARSED K79) has no Trp or Gly and is nearly all midsize mobile polars, so side-chain fit is not very diagnostic. The chain B N-terminus is now known to fold into a long helical extension of each Nsp8 copy when there is a long RNA transcript they can stabilize, as happens in the later PDB: 6yyt (24).

7btf also has the nine-residue sequence shift in Nsp12 described below for 7bv2, presumably inherited from the earlier PDB: 6nur and PDB: 6nus SARS-CoV structures at 3.1 Å (25).

#### Other peptide flips and CaBLAM outliers

ISOLDE does not yet look at CaBLAM outliers explicitly, but it made a number of peptide “flips” (rotations of 90–180°), which are usually associated with CaBLAM outliers. Fig. 3 *c* shows an especially clear CaBLAM diagnosis in the Nsp12 chain at the end of the A717–734 helix. The problem is flagged by two CaBLAM outliers and a CaBLAM C*α* geometry outlier. A peptide flip of the 733 CO (red ball on the O) corrects all three outliers, makes an additional H-bond at the helix C-cap, and fits the density somewhat better (Fig. 3 *d*). Most peptide-flip CaBLAM corrections in the rebuilt structure are clear improvements. However, flip corrections attempted in broad, low, or patchy density are often ambiguous as to which version (or both, or neither) is preferable; such marginal changes were seldom made in the new version.

#### A thought for the future

**Figure 4:**
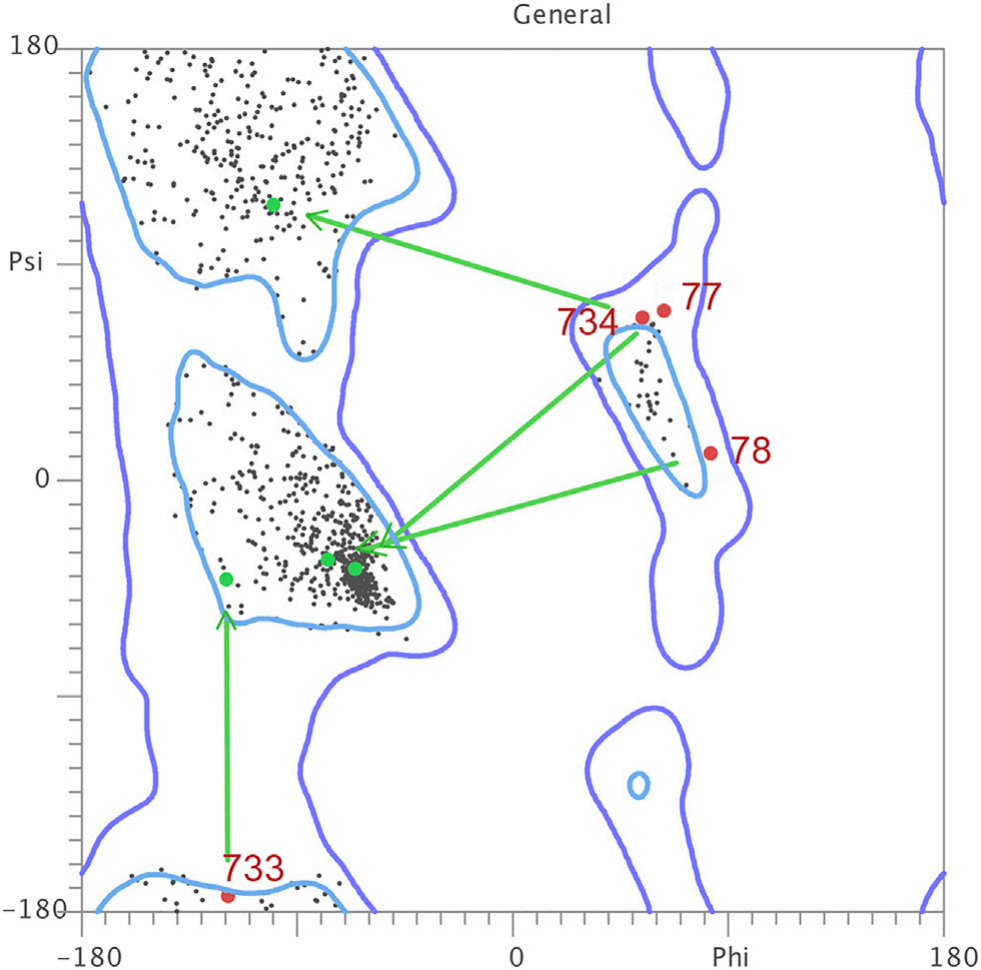
Ramachandran *ϕ*,*ψ* plot for 7btf, with original values for CaBLAM outliers as red points and green arrows to the corrected values, each of which moves to a different region of the plot. The four examples are on either side of the misoriented peptides for B77 and A733. To see this figure in color, go online.

It seems from the 7btf Ramachandran plot that *ϕ,ψ* values were restrained in refinement (diagnosed by too many points along the cyan contour separating favored from allowed values and a near-complete vertical cutoff at *ϕ* −60°). This helps keep refinement from diverging and, for instance, progressively distorting good secondary structures. It gives artificially good traditional Ramachandran scores but actually makes many of the conformations worse rather than better by pulling them into the wrong local minimum. This problem happens because the bumps for peptide CO oxygens disappear into the tube of backbone density somewhere between 2.5- and 3-Å resolution, so that badly incorrect peptide orientations are the most common type of misfitting by ≥3 Å (15). For each backward peptide, the preceding *ψ* and the following *ϕ* are very incorrect, so each of those Ramachandran points is usually close to the wrong local minimum. Fig. 4 shows this for two cases of 7btf CaBLAM outliers: at the B76 awkward return to correct sequence register (Fig. 3 *a*) and at the A733 helix terminus (Fig. 3 *c*). The points for 7btf are in red, always very close to the favored contour (cyan), with a green arrow pointing to the better, rebuilt answer, always in a quite different part of the Ramachandran plot. The preferred strategy (not always possible in rushed circumstances such as this) is to model regular secondary structures initially, to fix as many CaBLAM outliers as feasible before refinement, and then to restrain H-bonds rather than Ramachandran values.

A reimplemented tool called the Rama-Z score (26) is sensitive, especially for very large structures—not only to those with many Ramachandran outliers but also to structures refined with simplistic Ramachandran restraints, a very useful diagnostic. However, refinements are now beginning to apply the same criteria as in Rama-Z; that enables them to score well on Rama-Z even with restraints applied but, unfortunately, still makes the underlying problems worse rather than better unless they have been fixed beforehand.

### RNA-dependent RNA polymerase complex with RNA and remdesivir

The cryo-EM structure PDB: 7bv2 has 50 nucleotides of primer-template RNA helix bound, along with the potentially therapeutic remdesivir inhibitor (27). The resolution is unusually good at 2.5 Å, and the accompanying PDB: 7bv1 apo structure at 2.8 Å provides a close comparison. We, and many others, greatly appreciate the rapid deposition of these important structures and their maps. The 2.5-Å resolution provides quite clear density for both backbone and side chains, especially in the central core of the particle. At the other extreme, as typical for some of the outer regions and chain termini, the map density is so weak, patchy, confusing, or missing altogether that it does not effectively determine a most-probable conformation. In between, however, are regions where local mistakes can happen that are reliably correctable on close analysis.

#### Some RNA conformations are conformational outliers with poor density fit

**Figure 5:**
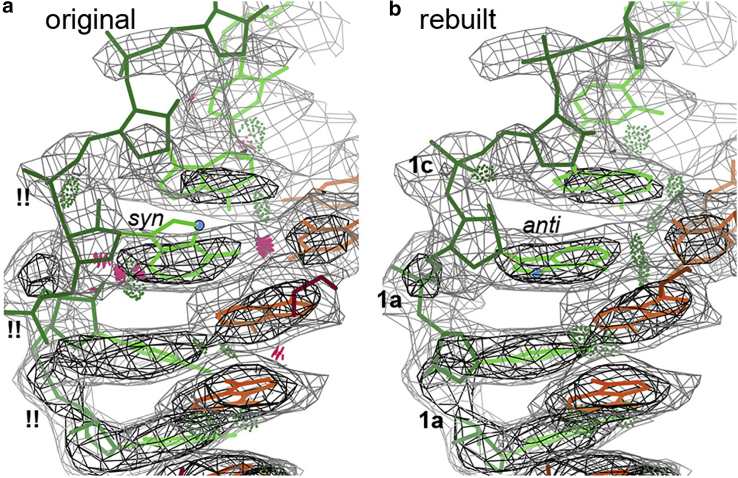
(*a*) Bad base and backbone conformations in RNA product strand. (*b*) Rebuilt, with better density fit. To see this figure in color, go online.

Most of the RNA in 7bv2 forms a regular A-form double helix with very strong basepair density. However, as shown in Fig. 5 *a*, template-strand (T) nucleotides 17–19 are modeled with **!!** outlier backbone conformers (11), the T A18 base is in the unusual *syn* orientation with clashes, and all three fit quite poorly to the clear density. This problem was corrected during the rebuild in ISOLDE and was confirmed using MolProbity’s ribose pucker, RNA-suite conformer, and all-atom-contact functions (7), along with examining by eye the fit of model to map. For the rebuild in Fig. 5 *b*, the density fit is excellent in backbone conformers A-form **1a** (and a close **1c**) with no clashes and better basepair H-bonding. At T 20 and above, the RNA helix makes no protein contacts, the density rapidly deteriorates, and neither model is convincing. Probably, that part is mobile and no one conformation fits the fragmented map. However, the primer and template strands are entirely complementary, with a G•C pair at the far end, and they will not have unfavorable conformations where there are no contacts to force strain. Ideally, they would be modeled as two or three copies of A-form, gently bending or twisting. The later 6yyt structure (24) has now shown that longer RNA product adopts very regular A-form, stabilized by long *α*-helical extensions folded from the two Nsp8 N-termini.

From the T 16 and P 15 basepair down to the active site in 7bv2, both protein and RNA look very good, until the single-stranded end of the template, where the U9 to U10 suite is clearly quite extended but the T 9 base and all of T 8 are largely disordered.

#### Ambiguities in the active site

**Figure 6:**
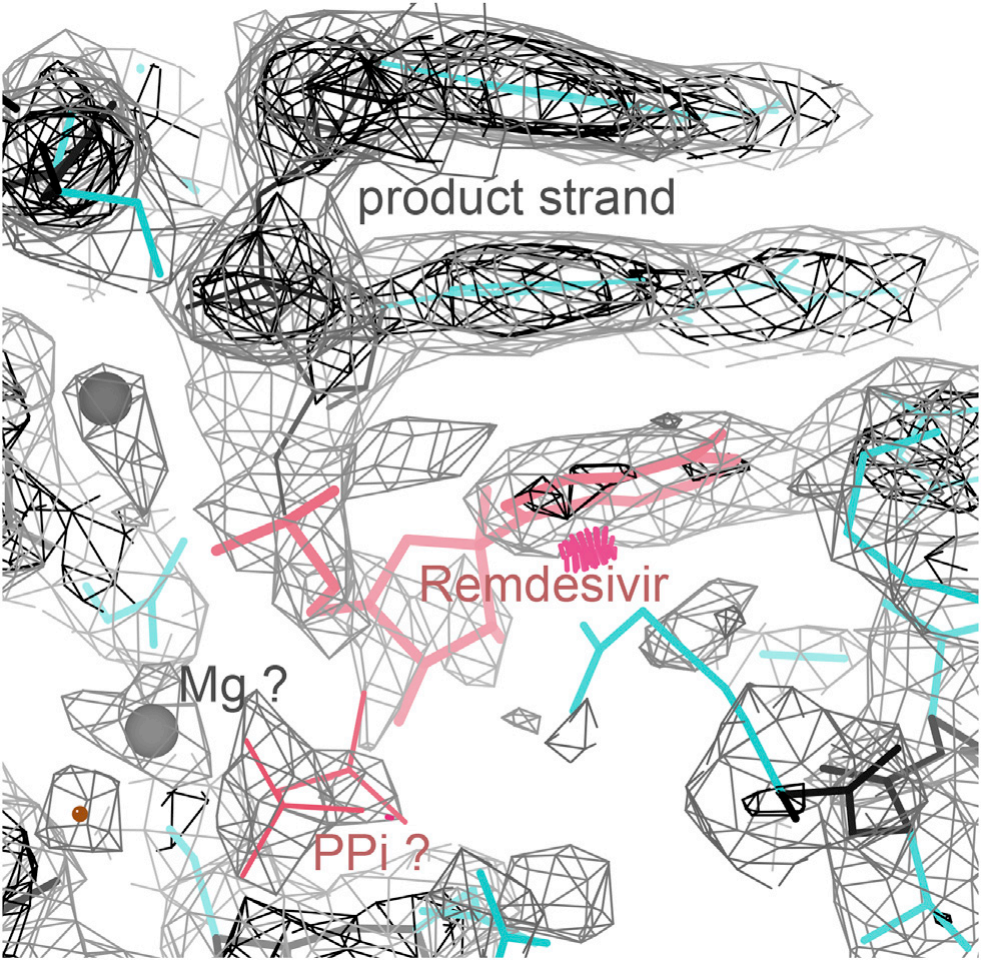
Remdesivir at half occupancy: highest density ∼9*σ* versus highest density of neighbor basepair at ∼18*σ*. The active site is at front left. To see this figure in color, go online.

The remdesivir is well stacked and base paired in the ligated product monophosphate form, as modeled (Fig. 6). It fits the density well, but that density is only strong enough to account for about half occupancy, which implies that only about half of the cryo-EM particles have remdesivir covalently bound. It is therefore not surprising that the adjacent active-site space has very low, patchy density that presumably represents some mixture of ligated and unligated states. The modeled Mg ions, pyrophosphate, and waters may be part of that mixture but not at high occupancy in any one position. We made one clear Mg-to-water correction at Mg A1006 but could not produce a clean model in the active site.

#### Sequence −9 register shift in the isolated C-terminal fragment of nsp12

**Figure 7:**
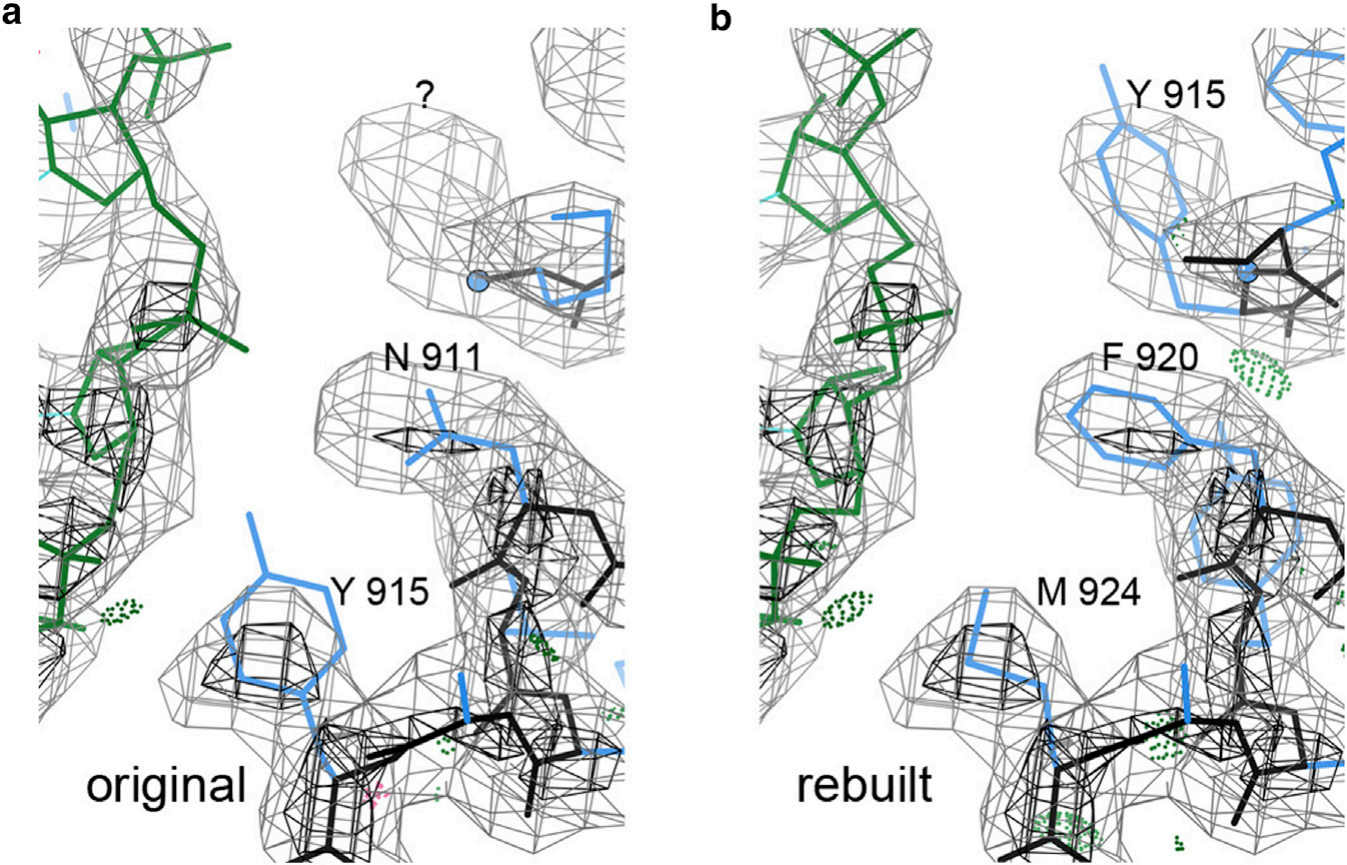
Sequence offset by nine residues. (*a*) Poor density fit in 7bv2. (*b*) Offset corrected, with excellent density fit. To see this figure in color, go online.

This isolated stretch of model lies between the C-terminus and an unmodeled gap following A895. The misalignment problem has been inherited from 6nur through 7btf and now to 7bv1 and 7bv2. In each case, it extends across however much of the fragment was modeled. The potential sequence contains four large aromatics (Tyr915, Trp916, Phe920, and Tyr921) whose fit to their side-chain density is highly diagnostic. In these misaligned regions, Met906, Leu907, Asn911, and Thr912 are much too small for their clear, connected side-chain densities, which can be beautifully filled by the four aromatics. Fig. 7 shows both the proximity of the RNA (green) and the badly filled aromatic side-chain densities. In 7bv2 at left, the line of three side chains up the center is Tyr915, which is a bit too big and the wrong shape for that density; Asn911, which is too small; and Met 906, which does not get into the density at all because of a side-chain-backbone switch around its C*α* (blue ball on the backbone N), which prevents fitting of a few earlier residues with density. In the rebuilt model at right, Met924, Phe920, and Tyr915 fit perfectly. In this view, the end of Trp916 fits the density in the top-right corner.

**Figure 8:**
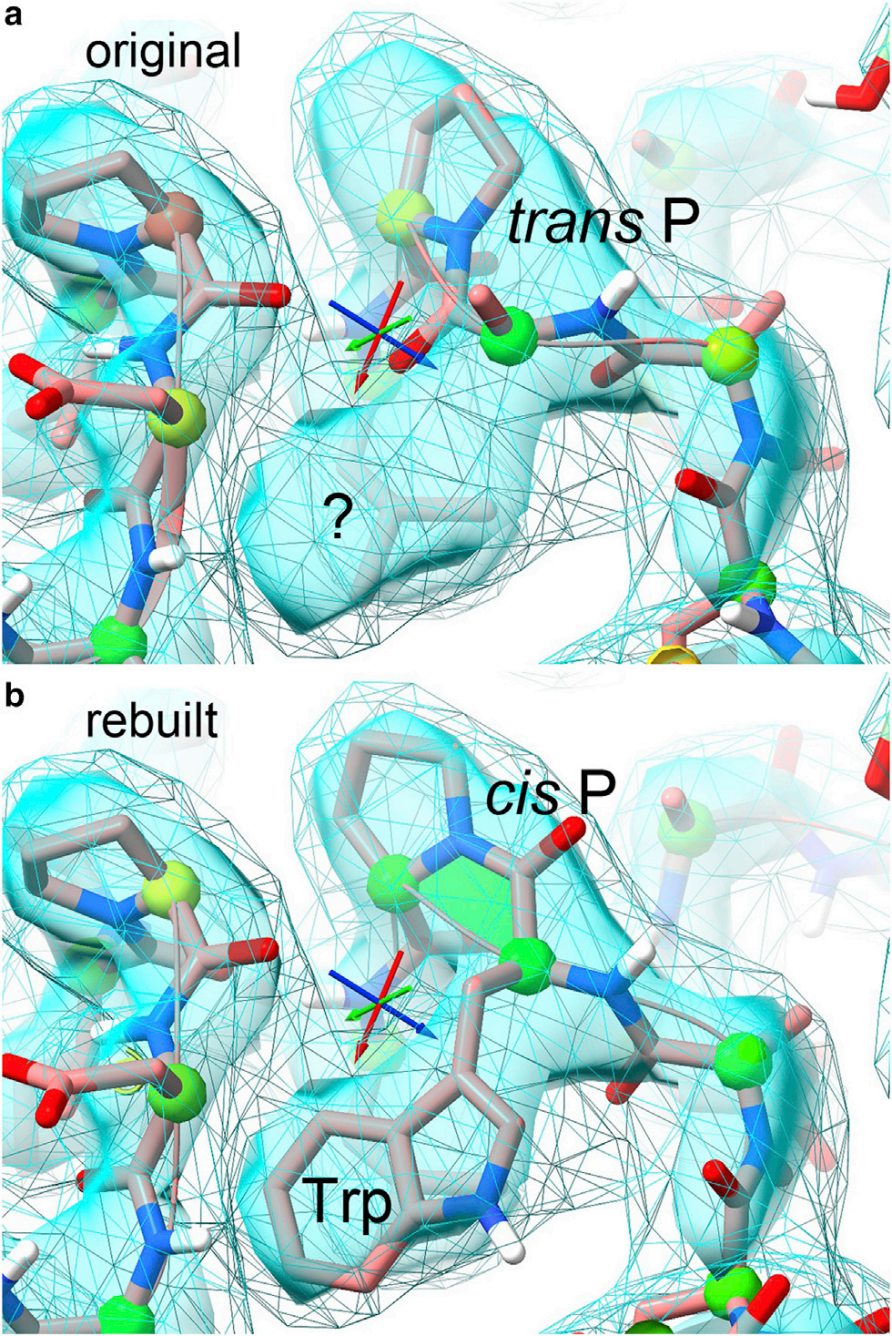
Making a correction in ISOLDE. (*a*) Pro B183 was forced to be *trans*, distorting the model so much that Trp182 cannot get near its obvious side-chain density. (*b*) When rebuilt as a *cis* peptide, all map fit improves, and the Trp occupies its density. The transparent cyan surface is original map density; wireframe is map smoothed by +50 in B-factor. N and O atoms are color-coded; spheres on C*α* atoms code Ramachandran quality; green trapezoid flags the *cis* Pro peptide. To see this figure in color, go online.

Unfortunately, current real-space correlation measures are sensitive only to atoms with no density, not to density with no atoms, so they do not detect sequence misalignments well. Because the sequence does not go back into register at either end of the offset, there are no awkward backbone compensations for CaBLAM to detect either. However, by visual examination, the −9 shift is unambiguous once considered as a possibility. This is worth correcting because it moves residues by extremely large distances and also because three of its residues are at the interface with RNA.

#### All prolines were modeled as *trans*, but two of them should be *cis*

Only ∼1 in 3000 nonproline peptides are *cis* (14), and indeed they should probably never be fitted that way at 2.5 Å and certainly never at 3 Å unless known from other data. But ∼5% of prolines are *cis*, so they are relatively common, and the Pro ring makes the distinction much more evident in the map density. It seems that the modeling process used for 7bv2 went overboard and forced all peptides to be *trans*, not just all non-Pro. That was the wrong answer in two cases. Pro B183 (Fig. 8 *a*) is especially bad as *trans*. The Pro itself is a poor fit to density (top center) and has both a CaBLAM outlier, which indicates a peptide orientation incompatible with the local C*α* trace (7), and a C*α* geometry outlier. Most tellingly, the preceding residue is distorted so much that its Trp side chain cannot get anywhere near its gorgeous, unoccupied density (bottom center) and was fit as just a C*β* stub. When the Pro is changed to *cis* in ISOLDE and the conformation relaxed, then the Trp slides easily into that classic side-chain density (Fig. 8 *b*).

#### Other peptide flips and CaBLAM outliers

**Figure 9:**
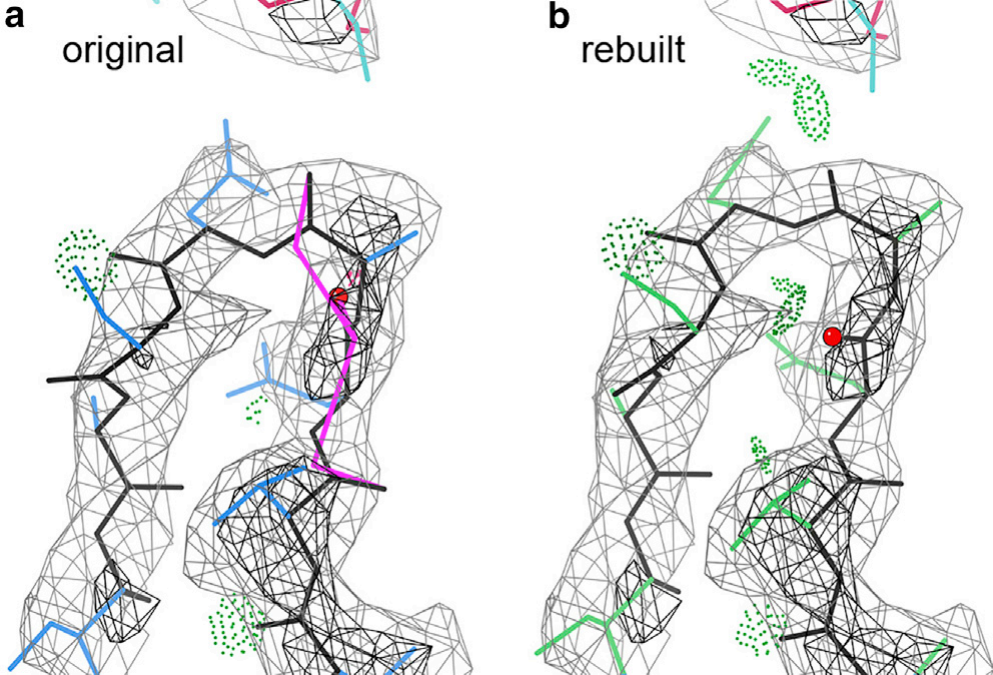
(*a*) Two adjacent CaBLAM outliers (*magenta*) in 7bv2 chain B. (*b*) Rebuilt as a tight turn, by flipping the CaBLAM-central CO orientation ∼140° (*red ball* on O). To see this figure in color, go online.

Our rebuild made a number of peptide flips in 7bv2 (rotations of more than 90°), almost all of which are associated with CaBLAM outliers. Fig. 9 *a* shows a clear example in chain B (Nsp8) in a *β*-hairpin loop. The problem is flagged by two successive CaBLAM outliers. In Fig. 9 *b*, a peptide flip of the central 161 CO (red ball on the O) corrects both outliers, fits the density somewhat better, forms a tight turn, and makes four more H-bonds, two across the turn and two that bridge to chain C (Nsp7, at top).

Peptide flips and rotamer changes matter most if they are in important places, such as near the active site or in a chain-chain interface, but should always be corrected if the new version is unambiguously better. However, one should remember that CaBLAM outliers are declared at a score contour level that excludes 1% of the quality-filtered reference data, so as many as 1% of the outliers may in fact be correct. A possible example is the Gly A678 CaBLAM C*α* geometry outlier near the active site; it is in an unusual Pro-Gly-Gly sequence, seems to be fit correctly, and has tight local contacts that prevent building it differently. Because it immediately precedes Thr680 in the active-site area, it might be one of the cases that conserves a less favorable but genuine outlier conformation because it better supports biological function.

### Depositor re-versioning as an efficient route to improved structures

Before the PDB News announcements of July 31, 2019 and February 18, 2020, if the “depositor of record” (the principal investigator) for a structure later found a need to change its atomic coordinates, sequence, or chemical description (but still from the same data), they had to obsolete it by an updated model with a new PDB code. Understandably, that process was only invoked for really serious reasons. Now, there is an archival versioning system for PDB codes that jumps by 1.0 for major changes as above (depositor-initiated) and by 0.1 for formatting or other minor changes (usually done by the wwPDB itself). Fig. 1 *b* shows this in the revision history for 6vyo, in which version 2.0 was the Zn-to-Cl ligand identity change. Previous versions of a structure can be accessed from a separate, versioned FTP archive at the PDB. This new process has been invaluable for immediate availability of new SARS-CoV-2 structures, allowing further checkouts and changes to proceed easily and to propagate immediately into new database downloads even before publication.

As well as providing our own rebuilt models on the CSTF site (similar to what is done by PDB-Redo, the covid-19.bioreproducibility.org site, and probably others), we have taken advantage of the wwPDB re-versioning system to alert depositors to the few most urgent and clear changes in their SARS-CoV-2 structures, encouraging them to make and confirm those changes themselves in their own model for rapid deposit of an improved major version with the same PDB code. This process has typically taken only somewhat over a month between initial and re-versioned releases (∼2 weeks for us to find and convincingly document problems and 2 weeks for depositor change and version release). Besides direct responses to our emails, such as for 6vyo, 7bv1, and 7bv2, information about major changes such as sequence misalignments sometimes propagates via the grapevine, such as for 6m71 and 7btf. Once an early structure has been re-versioned, later structures of the same molecule will usually start from the improved model, whether solved by the same or by different groups (Table 1). For the RNA polymerase complex, that was true for five of six newer structures: explicitly stated in the paper for 6yyt (24) and the paper for PDB: 7bzf and PDB: 7c2k (28); true also for PDB: 7ctt and PDB: 7aap, but not for PDB: 6xqb.

**Table 1:** PDB entries discussed in this paper, grouped by molecule

PDB ID	Method	Res. (Å)	Rel. date^a^	Molecule/title	Proteins	RNAs	Ligands (in v1.0)	Problem	Major rev.^a^	Ref.
6nur	cryoEM	3.10	05/29/19	SARS-CoV RdRp^b^	Nsp12, Nsp8, Nsp7	-	Zn	seq offset	-	(25)
6nus	cryoEM	3.50	05/29/19	SARS-CoV RdRp	Nsp12, Nsp8, Nsp7	-	Zn	seq offset	-	(25)
6vyo	x-ray	1.70	03/11/20	nucleocapsid Pprot	4 x RNA-bdg dom	-	Zn	Zn for Cl	04/08/20	-
v2.0	x-ray	1.70	04/15/20	nucleocapsid Pprot	4 x RNA-bdg dom	-	Zn, Cl	v2.0 OK	-	-
6w41	x-ray	3.08	03/25/20	spike/antibody	spike RBD, Fab	-	NAG, SO_4_	bad SS, NAG	-	-
6yLa	x-ray	2.42	04/15/20	spike/antibody	spike RBD, Fab	-	NAG	hi-res OK	-	-
6w9c	x-ray	2.70	04/01/20	PL protease	3 x protease	-	Zn, Cl	bad ZnFs	05/06/20	-
6wrh	x-ray	1.60	05/06/20	PL protease	protease	-	Zn, Cl, PO_4_	hi-res OK	-	-
6m71	cryoEM	2.90	04/01/20	RdRp	Nsp12, Nsp8, Nsp7	-	-	seq offset	05/27/20	(23)
7btf	cryoEM	2.95	04/08/20	RdRp complex	Nsp12, Nsp8, Nsp7	-	Zn	seq offset	05/27/20	(23)
7bv1	cryoEM	2.80	04/22/20	RdRp apo complex	Nsp12, Nsp8, Nsp7	-	Zn	seq offset	05/27/20	(26)
7bv2	cryoEM	2.50	04/22/20	RdRp/RNA/RTP	Nsp12, Nsp8, Nsp7	30, 20	remdesivir, Zn, Mg, PPi	offset, RNA	05/27/20	(26)
6yyt	cryoEM	2.90	05/13/20	RdRp/RNA product	Nsp12, Nsp8, Nsp7	4 × 18	Zn	-	-	(24)
7bzf	cryoEM	3.26	06/03/20	RdRp post-catalytic	Nsp12, Nsp8, Nsp7	31, 14	Zn	-	-	(27)
7c2k	cryoEM	2.93	06/03/20	RdRp pre-catalytic	Nsp12, Nsp8, Nsp7	29, 18	Zn	-	-	(27)
6xqb	cryoEM	3.40	07/29/20	RdRp/RNA	Nsp12	2 × 9	Zn, Mg	seq offset	-	-
7ctt	cryoEM	3.20	10/02/20	RpRp/favipiravir	Nsp12, Nsp8, Nsp7	40, 20	favipiravir, Zn, Mg	-	-	(28)
7aap	cryoEM	2.60	10/23/20	RpRp/favipiravir	Nsp12, Nsp8, Nsp7	30, 24	favipiravir, Zn, Mg	-	-	-

a Dates compare release with revision dates; a major revision changes coordinates, sequence, or chemical identity and moves to the next integer version.

b RdRp = RNA-dependent RNA polymerase. All structures are from SARS-CoV-2 except the first two.

## Conclusions

In response to the pandemic, structural biologists worldwide have responded with unprecedented speed to solve the structures of SARS-CoV-2 proteins and complexes and have broken precedent to deposit and release those structures immediately for the benefit of further COVID-19 research and development. There has been some criticism of these early releases and of the posting of COVID-19-related research on preprint servers such as bioRxiv as undesirable shortcutting of the peer review system. We believe these complaints have missed the very positive aspects of what is actually going on. Most of these initial releases and preprints will eventually go through the standard peer review process to achieve formal publication, and many have by now already done so. In the meantime, they have gone through a much stricter scrutiny and review than normally possible, done by the entire community. That is exemplified by the work described in this article, which has also rippled into improving related later structures.

Immediate coordinate release of an initial, preliminary model seems desirable only in urgent circumstances like the present—normally, the depositors themselves should thoroughly check out their own structures. Release after checkout is certainly possible and has advantages, but would probably only seldom attract community validation and correction.

Perhaps the most important takeaway message from our work is that coordinates and density maps need to be, and should be, provided to reviewers in the structured environment of standard peer review, in which they would be enormously helpful to the review and any misuse of that information could be documented and censured. In current peer review for journals, only a validation report is supplied to the reviewer, not the coordinates or the map. Therefore, they cannot judge, as we have often been able to do here, whether an outlier is actually wrong or genuine and how much it matters to the reported conclusions. In standard peer review, sometimes the validation report prompts a request for more qualified wording of specific conclusions, but coordinates are almost never changed (we know of only one case, in which model and data were actually available to the reviewer). The most effective way to initiate this paradigm change of providing reviewers with coordinates and maps is for structural biologists, in our role as reviewers, to routinely request those data as a condition for doing the review.

The results presented here also demonstrate the great value of the wwPDB’s new archival versioning system, which enables the depositor of record to update to an improved model without changing the PDB code. This just-in-time facility has been invaluable for the SARS-CoV-2 structures, and going forward, it will encourage a general improvement in the accuracy of the database for the benefit of all users and uses, including more leisurely retroactive versioning as well as for urgent early releases.

A few of the early-release SARS-CoV-2 structures were accompanied by a short initial write-up as a bioRxiv preprint, such as for 6m71 and 7btf (29); that preprint explained their strategy on the issue of disulfides versus Zn sites that would otherwise have seemed like an error. Those preprints were very useful and missed when absent, and we would strongly encourage their provision for early releases, as well as for depositions for which formal publication is not planned. Conversely, preprint posting of a structure report should always be accompanied by deposition and release of coordinates and map(s) so that potential users of the information can fully check validity.

At a more specific and detailed level, we hope this work provides convincing evidence that (on the negative side), at the current state of the art, there are local model errors even in generally excellent x-ray or cryo-EM structures that cannot be fixed by downhill refinement. Yet, in compensation, on the positive side, most of those errors can be located even in very large structures by tools such as CaBLAM and are very often tractable to user-guided correction in rebuilding systems such as KiNG, Coot, Chimera, or ISOLDE. The cases treated in detail here can serve as guidance in strategies for diagnosis, identifying alternative fittings, and testing whether an alternative is a clear improvement.

## Author contributions

T.I.C. performed the model optimizations in ISOLDE, and J.S.R. made and evaluated corrections of CaBLAM, RNA, and other MolProbity outliers. C.J.W. and V.B.C. interacted closely with the CSTF and modified MolProbity and KiNG output to meet their website needs. J.S.R., D.C.R., and T.I.C. wrote and illustrated the emails to depositors and the initial draft of the article. All authors reviewed, edited, and approved the article in its submitted and revised forms.
